# HIV/AIDS awareness and risk behavior among students in Semey, Kazakhstan: a cross-sectional survey

**DOI:** 10.1186/1472-698X-8-14

**Published:** 2008-12-16

**Authors:** Marit Hansson, Leo Stockfelt, Marat Urazalin, Clas Ahlm, Rune Andersson

**Affiliations:** 1 Division of Infectious Diseases, Department of Clinical Microbiology, Umeå University Hospital, SE-901 85 Umeå, Sweden; 2 Department of Infectious Diseases, Sahlgrenska Academy, University of Gothenburg, SE-405 30 Göteborg, Sweden; 3 Semipalatinsk State Medical Academy, 490050 Semey, Republic of Kazakhstan; 4 Research and Development Centre, Skaraborg Hospital, SE-541 85 Skövde, Sweden

## Abstract

**Background:**

Until recently, young people in Kazakhstan have been only moderately affected by the global HIV epidemic. Today, however, the HIV epidemic in Central Asia is one of the most rapidly increasing epidemics in the world. It is mainly concentrated to vulnerable groups such as intravenous drug users, sex workers, the purchasers of sexual services and the financially marginalized. Young, sexually active people may however be the gateway for the epidemic to the general population, and knowledge about their attitudes and behavior is therefore important in planning preventive measures.

**Methods:**

To gather information about young students and their attitudes and knowledge about HIV/AIDS, we collected 600 structured questionnaires and made 23 semi-structured interviews among three groups of students. Response rate was 99%.

**Results:**

Almost 99% of the respondents had heard of HIV/AIDS, and 89% could identify ways to protect oneself against sexually transmitted HIV/AIDS. The main routes of transmission, sexual contact without condom and intravenous drug use, were both identified by 97% of the students. Twenty-five percent of the female students and 75% of the male students had had one or more sexual partners. More than 30% of the young men had purchased sex, and homosexuality was widely stigmatized.

**Conclusion:**

Risks for the spread of HIV/AIDS among young people in Kazakhstan include prostitution as well as stigmatization of the HIV positive and of homosexuals. Protective factors are good knowledge about risks and protection, and opportunities to talk and gather information about sexuality and HIV/AIDS.

## Background

The Republic of Kazakhstan is a large country with a relatively small population of 15 million people. It was formerly part of the Soviet Union and is now a multiethnic society with some 100 different ethnic groups, dominated by Russians and Kazakhs. The main religions are Islam, 47%, and Russian Orthodox Christianity, 44%, although it is a relatively secular country. Kazakhstan had some difficult years after the fall of the Soviet Union but now the economy is growing fast, with the GNP increasing by over 10% per year. The relatively stable political situation, the absence of armed conflicts and the vast amounts of natural resources all give hope for future economic progress [1]. Semey, or Semipalatinsk as it is called in Russian, is a city in northeastern Kazakhstan with approximately 293,000 inhabitants, one of the larger industrial cities in the country and long the centre for the Soviet nuclear weapon testing.

Central Asia and Eastern Europe still have low numbers of reported HIV cases, but since the year 2000 the growth rate of the epidemic has been the fastest in the world [2,3]. By 2001, an estimated 630,000 people were infected with HIV, and this rate increased by 150% until 2007, with 1,6 million people infected [4]. Factors creating a risk environment include injecting drug use (IDU), migration, economic decline, increasing unemployment and decline in the public health infrastructure [5]. HIV/AIDS affects young people more than other age groups, especially young men, and people living near subsistence level [6]. Central Asia is estimated to have 500,000 drug users, of whom more than half inject, and of the registered HIV cases in the region, over 75% were acquired through IDU [2]. However, sexual transmission is likely to increase in the near future, spreading the infection to a much larger population [7].

The HIV situation in Kazakhstan is mostly concentrated to highly vulnerable populations such as injecting drug users and sex workers, but the disease is also spreading to other vulnerable groups including youth, migrants, and truck drivers. Newly registered HIV cases increased from 699 in 2004 to 1,745 in 2006 [8]. WHO/UNAIDS estimates range from 11,000 to 77,000 HIV positive individuals [6]. Injecting drug use is the most significant route of HIV transmission in Kazakhstan today, but more than 25% of newly reported cases in 2004 were caused by unprotected sex. This indicates a shift in the state of the epidemic, spreading from high-risk groups into the general population, as is also happening in neighboring China [9].

The countries of Central Asia are likely to experience a serious crisis in terms of HIV/AIDS over the next 20 years, with development concentrated to injecting drug users, followed by a generalized epidemic with sexual transmission as the predominant mode [2]. Young sexually active people form a bridge population, through which the epidemic could spread from high risk groups, such as sex workers and intravenous drug users, into the general population [10,11]. Therefore knowledge about their attitudes and behavior is important when designing and implementing effective preventive measures. The aim of this study was to evaluate the knowledge, attitudes and risk behaviour among students in the city of Semey, a potentially important group in predicting and preventing an increase of the HIV epidemic in the Republic of Kazakhstan.

## Methods

### Study population

The study was performed in Semey, an industrial city situated in northeastern Kazakhstan with approximately 293,000 inhabitants. The Medical University of Semey, the four largest non-medical universities in Semey, and four out of 73 high schools in different parts of the city were selected to provide a representative picture of the attitudes and knowledge among students in Semey. With the approval of the university administrators, the investigators approached classrooms for volunteers. One third (34%) of the questionnaires were distributed at the Semipalatinsk State Medical Academy to medical students in years 1–7, another third (34%) to students in years 1–4 of four non-medical universities in Semey and the last third (33%) to 15 to 19-year-old pupils at four different high schools (Table 1). A total of 609 questionnaires were distributed, and all were returned. Nine of these questionnaires were excluded from the analysis; eight because a page was missing and one because the answers were obviously not serious. This makes the response rate 99%.

**Table 1:** Demographic Profile.

		High school students	University students	Medical students
Median age (years)		17	20	20
Gender (%)	Male	43	34	39
	Female	57	66	60
Ethnicity (%)	Kazakh	59	86	73
	Russian	31	10	14
	Other	9	3	13
Religion (%)	Moslem	62	89	81
	Orthodox	30	10	13
	Other	3	0	3

Men n = 252, women n = 348.

Out of the 600 students who answered the questionnaires, 23 volunteers were selected for semi-structured interviews, by asking for volunteers in the classes in which the questionnaires were distributed. The volunteers were asked to participate directly after answering the questionnaires, with an appointment later the same day in an undisturbed room. Of the individuals interviewed, 10 were men and 13 women. Eight were medical students, seven studied at other universities and eight were high school students. Ages ranged from 18–22, with a median age of 20.

### Ethical considerations

An ethical certificate for this study was not required [see Additional File 1]. Informed consent was obtained from the students, after we emphasized that participation in the study was voluntary, that they could stop participating at any time and that data would be presented so that identification at an individual level would not be possible. The respondents were also told that they did not have to answer any questions they found intrusive or embarrassing.

### Structured questionnaire

The self-completed questionnaire consisted of an introduction, a personal details section (sex, age, discipline, educational level, ethnicity, religion, and civil status) and questions covering four topics: knowledge of sexual health and HIV/AIDS, attitudes towards and experience of sexual activities, demands for reproductive health care services, and knowledge of the HIV situation in Kazakhstan.

### Semi-structured interviews

The semi-structured interviews were focused on the students' personal behavior and attitudes and their views on the situation for young people in Semey. The questions were clustered around the areas of sexuality, having HIV, special groups affected, and HIV in Semey, using mainly open questions. The interviews lasted on average 45 minutes and were performed by the investigators, with the interviewer having the same sex as the interviewed subject to minimize inhibition. When possible, the interviews were held in English without an interpreter, but in eight of the interviews an interpreter was used. The interviews were tape recorded and later transcribed for analysis.

### Statistics

Descriptive statistics such as frequencies, means, confidence intervals and ranges were used to summarize the data and compare the different groups according to educational level and program, ethnicity and sex. If the 95% confidence intervals were not overlapping, the differences were regarded as statistically significant.

## Results

### Information and knowledge

**Figure 1:**
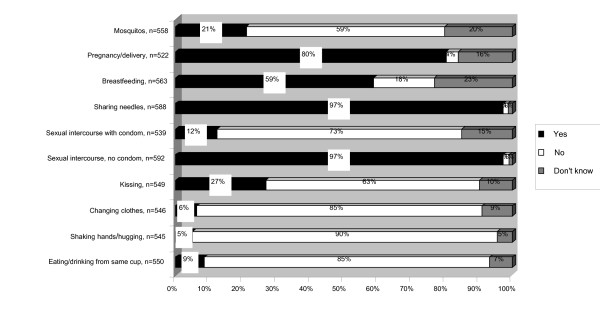
**How can HIV/AIDS be transmitted from one person to another?** Knowledge about routes of HIV transmission.

Almost all the respondents to the questionnaires, 99%, had heard of HIV/AIDS, but only 54% answered that there is a difference between HIV and AIDS. They were asked to choose ways that HIV/AIDS can be transmitted from a set list in the questionnaires (Fig 1). There was a clear consensus that sexual intercourse without a condom and sharing needles while injecting drugs are ways in which HIV/AIDS can be spread. From mother to child during pregnancy and delivery and breastfeeding were somewhat less often recognized as routes of transmission. Some answered that HIV can be transmitted by kissing, through mosquitoes or through sexual intercourse using a condom. Daily domestic contact was less commonly thought to transmit the disease.

Of the medical students who responded to the questionnaire, 76% answered that there is a risk of HIV spreading among the patients in a hospital, and 79% also thought that this could be prevented. Suggestions concerned correct haemotransfusion, checking blood from donors and not reusing syringes. In the interviews it was also often claimed that there is a risk of spreading of HIV in hospitals. A female university student said about the health care in Kazakhstan: 'nurses sometimes use one needle twice, maybe three times, because there is not enough money for personal injections.'

**Figure 2:**
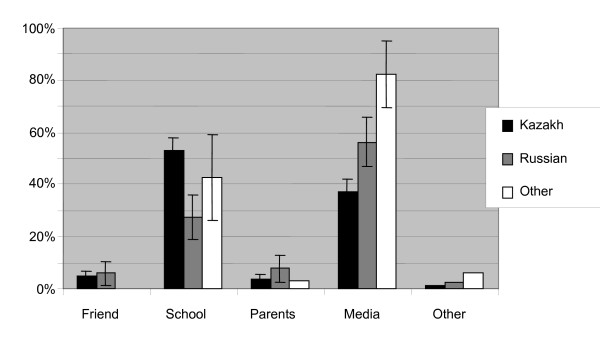
**Where did you first hear about HIV/AIDS?**. Source of first information about HIV. Kazakh n = 437, Russian n = 108, Other ethnicity n = 35.

The mean age for learning about HIV/AIDS was 13.1 years old. Many students expressed during the interviews thar it is important to inform children about HIV/AIDS from a young age, saying that they themselves had received the information too late. When asked in the questionnaires about their first source of information (Fig 2), school and media were the most frequent answers while only a few mentioned friends, parents or other sources. In contrast, many said in the interviews that giving information is the parents' responsibility and it was common to have received some information from the parents. For example, one female medical student said: 'I got information from my mother, and boys should get information from their fathers.'

The ethnic Kazakh students learned about HIV later than the ethnic Russian students, and their first source of information was more often school, whereas the ethnic Russian students more often learned from media and their parents.

**Figure 3:**
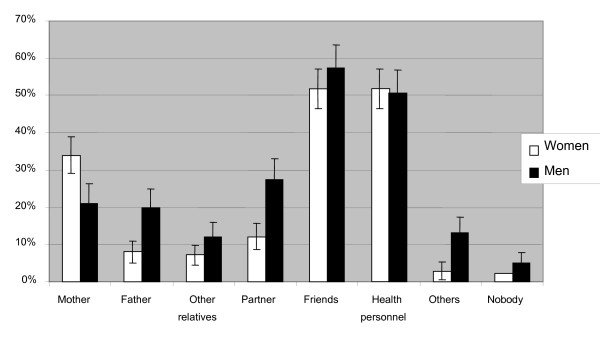
**With whom can you talk about HIV/AIDS?** People with whom to talk about HIV/AIDS. Men n = 251, women n = 347.

Most of the respondents had someone they could talk to about HIV and AIDS (Fig 3). Most often they could talk to friends and health personnel while fewer could talk to their partners or parents. Women could more often talk to their mothers and men more often to their fathers. This pattern was supported during the interviews, where female students often expressed that they preferred talking to other women, while male students preferred other men. Most students said in the interviews that they did not find it difficult to talk about sex, especially not with someone of the same sex and age.

### Attitudes toward HIV positive people

Only 6% of the respondents would want to have children if they knew they were HIV positive, while 77% answered no, specifying that the child might become infected, and 17% did not know. Nine people did not answer this question. There were no significant sex differences.

When asked in the interviews what would happen if the respondent fell in love with an HIV positive person, the concern was rather the risk of not being able to have children than the risk of becoming infected themselves. As one high school girl put it: 'Family is very important and joyful in our life. If you do not have a family, it is very terrible.' It was considered impossible to have a family with an HIV positive person. One female medical student said: 'I understand that with this person I can't create my family because it is dangerous for me and for my future children.' Almost all the interviewees concluded that because of the risk of becoming infected and not being able to have children, they would try to stop being in love with someone who was HIV positive.

In the interviews, we asked what would happen if the students themselves became HIV positive. Most said they would go to the doctor for advice and treatment. Some said they would die very soon and reflected about this, and generally the idea was that people with HIV lived only a very short time. Some of the students stressed that they would only tell people close to them, primarily their parents and family, for fear of being badly treated in the community. All of our interviewed respondents said that they would help a friend who was HIV positive, and one female university student said: 'In my opinion we shouldn't blame them because anyone could find themself in a similar situation'. The students seemed to think that although they would not treat a friend or acquaintance differently, other people would. In the words of one high school boy: 'Society will avoid them. But we must help them.'

### Attitudes toward testing and protection

Of our respondents to the questionnaires, 89% answered that there are ways to protect oneself against sexually transmitted HIV/AIDS. More of the men, 94% (95% CI 91–97), answered that it is possible to protect oneself, as compared with 86% (95% CI 82–89) of the women. The most common specified means of protection by far was to use condoms, though concern was raised over the quality of the condoms both in the interviews and questionnaires. In the interviews, the respondents said that although they knew that condoms are necessary, they still might not use them, especially if they had other means of birth control. For example, one female university student said: 'most girls are on the pill, because our boys don't think about using condoms, and the girls think only about the risk of getting pregnant.'

The majority answered that both partners have equal responsibility for making sure that a condom is used during heterosexual intercourse (Table 2). The women more often answered that both partners have equal responsibility, while the men more often answered that it is the man who has the greater responsibility. A female student working part-time in a shop said in her interview that she only sees boys come in to buy condoms and another female student said that it would be embarrassing for a girl to buy condoms.

**Table 2:** Responsibility for condom use during heterosexual intercourse, according to sex.

	Male students	95% CI	Female students	95% CI
Both	63%	57–70%	78%	74–83%
Men	31%	25–38%	19%	14–23
Women	5%	3–8%	3%	1–5%

Men n = 226, women n = 313.

Only one fourth of the students (26%) wanted to be tested for HIV, while 67% did not want to and 7% did not know. Eleven people did not answer this question. The most common reasons for not wanting to be tested were not having had any sexual partners or being sure of not being HIV positive. Reasons for wanting to be tested were 'just for interest' or 'to be sure'. The last question in the questionnaire, 'is there anything you would like to add?', was often answered with that there should be large-scale testing for HIV. Many respondents also talked in the interviews about getting oneself and one's partner(s) tested regularly.

### Sexual behavior

**Figure 4:**
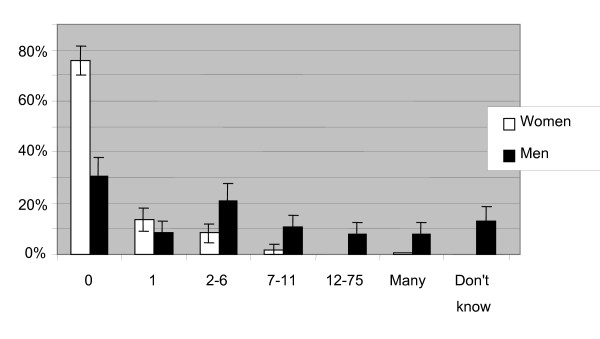
**How many sexual partners have you had?** Number of sexual partners. Men n = 151, women n = 216.

Among female students, 75% had not yet had a partner, while among male students this number was 25% (Fig 4). Of those who had had sex, the median number of sexual partners was five for the men and one for the women. The men also had a larger range of 0–25 sexual partners (with one outlier who had had 75 sexual partners) as compared with the women, 0–11 sexual partners. The men not only claimed to have had a larger number of sexual partners, they were also earlier in their sexual debut. The range for age of first sexual contact for men was 11–20, with a median of 16. For women the range was 14–22 with a median of 18. Since more than half of our respondents had not yet had a sexual partner, the actual median age of sexual debut will, of course, be higher.

During interviews, the importance of love in a sexual relationship was often stressed. Among the men, some said that it has been considered cool for men to have sex with many women, but that the trend has now changed and that the idea now is that sex belongs in a steady relationship and only when you are in love. Many of the women said that they would only have sex in marriage and the general view was that it was more acceptable for a man to have many sexual partners than a woman.

Very few students answered that they had had sexual relations with a partner of the same sex. Homosexuality was considered very shameful and described as a mental illness. Homosexuality was said to be very uncommon in Semey, and less common in Kazakhstan than in other countries. Although they did not associate this with the difficulties of being openly homosexual in Kazakhstan, many, such as one female medical student, said 'It would not be possible to be openly homosexual in Kazakhstan. People would laugh.'

A large number of the men, 32%, answered that they had purchased sex, but none of the women. Of the sexually active men 41% had purchased sex. This group was slightly older than the mean, 18.9 years as compared with 18.7, but this was not statistically significant. Of the male medical students, 39% (95% CI 33.5–44.5) answered that they had purchased sex as compared with 33% (95% CI 26.4–39.6) of the male university students and 25% (95% CI 20.6–29.4) of the male high school students.

In the interviews, opinions about prostitution seemed to be quite permissive. It was considered negative more because of the risk of transmitting sexual diseases than on the basis of moral standards. The attitude was that prostitution exists, is a tradition and cannot be stopped. Most of the men but few of the women we interviewed said that there was a lot of prostitution in the city. Street prostitution was not described, but rather hotels and saunas where men would go to celebrate birthdays and other events. One male student mentioned male prostitution, otherwise only female prostitution was discussed. Two of the interviewed male students said openly that they had been to prostitutes several times. Both had stopped now, one because of the risks of infection, the other because he only wanted to have sex with his girlfriend.

### Drug use

Drug addicts were considered to be at risk of HIV infection by 58% of the students. The respondents were generally aware that it is important to avoid sharing injection equipment for preventing the spread of HIV. Many of the interviewees talked about needles as a route of HIV transmission in Kazakhstan, and said that they see many drug users. Several of the interviewed students described friends, or more commonly former friends, who used drugs. A few admitted to having tried smoking cannabinoids, but had stopped for health reasons. None admitted to injecting drug use, and the students were generally negative to drug use. Most said that drug use is common and increasing, both among younger and older people.

### HIV in Kazakhstan

Of our respondents, 43% answered that there is a risk of a future HIV epidemic in Semey and Kazakhstan, while 16% did not believe in such a risk. Forty-one percent did not know, and nine students did not answer. Frequent arguments for why there is a risk were high levels of drug addiction and prostitution. Young people were often described as having a weak moral stance, with a number of sexual partners and an uncontrolled sex life. Frequently the answers concerned insufficient levels of information and knowledge. A few respondents pointed out foreign people, uneducated people from rural areas, or poor and homeless people as factors affecting the spread of HIV.

Reasons why there would not be a risk of an HIV epidemic in Semey/Kazakhstan were that measures are being taken to prevent the spread of HIV and AIDS, and that information is available. Religion and public behavior in Kazakhstan were said to have a protective influence, and the limited number of HIV positive people in Kazakhstan was also mentioned as a hopeful factor.

## Discussion

Almost all the students had heard of HIV/AIDS, which indicates that their basic awareness was good. On the other hand, only half of the respondents answered that there are differences between HIV and AIDS, indicating a lower level of deeper knowledge. The respondents were good at giving the correct answers to the routes of HIV transmission, 'sexual intercourse without a condom' and 'sharing needles for injecting drugs'. These are also the most important routes of transmission for the general population to be aware of. They found it more difficult to exclude incorrect routes, which has also been seen in several other studies [12-14]. Daily domestic contacts were usually excluded, but kissing and sexual intercourse with a condom were often considered as routes of transmission. These misconceptions may increase the stigmatization of HIV positive people, as was shown in a study in San Francisco, California where students with misconceptions about transmission of HIV through casual contact were more likely to answer that students with AIDS should not be allowed to attend school [15].

The medical students were aware of the risks of HIV spreading between patients in a hospital and had many suggestions about how to prevent it. As was recently seen in the outbreak of transfusion-related HIV among children in Kazakhstan [16], these are risks of which medical students need to be aware, and it is a positive sign that they had many ideas about how to prevent this.

Schools and the media were the main sources of information about HIV/AIDS for our respondents. In the interviews, many claimed to have received information from their parents, but this was not supported by the data from the questionnaires. Most of the students had at least someone they could talk to about HIV and AIDS, most often friends and health care personnel. The ethnic Kazakh students learned about HIV/AIDS later than the ethnic Russian students, and their first source of information was more often school. It seems that the ethnic Russian students, at the time information was given at school, had already heard about HIV from elsewhere.

When asked what the students would do if they were diagnosed with HIV, most said that they would tell their parents and go to the doctor. There was not the tendency to secrecy that was seen in the nearby Chinese province of Xinjiang, where some of the medical students said that they would go to another city to get treatment [17]. Confidence in the health care personnel is important so that people will get themselves tested if they think they may have been infected. There was good knowledge about the use of condoms as a way of preventing transmission of sexual diseases, but many of the interviewees admitted that they might not use them anyway. Almost three out of four answered that both partners have equal responsibility to make sure that a condom is used during heterosexual intercourse. Although it seems that in reality the man has more responsibility for the buying and use of condoms, it is positive that the common opinion is that both partners are equally responsible. Only one fourth of the students wanted to be tested for HIV, often motivating their reluctance with not having had any sexual contacts or not being in the risk zone for infection.

The men reported a larger number of sexual partners and an earlier sexual debut. These results may be influenced by the common and accepted habit of young men to go to prostitutes. That one third of the men had purchased sex is a remarkably high number, especially considering the young age and relative sexual inexperience of this group. The number of students purchasing sex shows that prostitution is a possible gateway for the HIV epidemic to spread to the general population. Approximately 40% of female intravenous drug users in Central Asia are engaged in sex work [18], and data from Sexually Transmitted Infection (STI) Clinics in Kazakhstan show that 75% of female sex workers have at least one STI [19].

Stigmatization of homosexuals is a problem and possible risk factor among students in Semey. Their stigmatization may lead to difficulties in reaching this group with information, and to their partial exclusion from testing and health care. Several studies from countries such as Russia [20], China [21], and Bulgaria [22] indicate the importance of targeting men who have sex with men in HIV prevention, which may be difficult in Semey owing to this stigmatization.

Drug use is said to be common and increasing, and to occur both among students and older people. None of the students in the interviews admitted to having injected drugs, although some had tried lighter drugs. In Xinjiang this subject was mentioned less often. The importance of drug use in the transmission of HIV is highlighted in several studies from countries such as Russia [23], Georgia [24], and Ukraine [25], and drug use is claimed to drive the HIV transmission curve upward in Central Asia [26]. It seems, however, that in this group of quite ambitious young students, prostitution is a bigger risk factor than drug use in the spread of HIV.

Approximately 40% of our respondents answered that there is a possibility of a future HIV epidemic in Kazakhstan. The most common reasons mentioned, in line with country estimates, were high levels of drug addiction and prostitution. Protective factors mentioned were actions that is taken to prevent the spreading of HIV/AIDS, existing information, religion, and the behavior of the people of Kazakhstan, as well as the limited number of HIV positive people today.

### Limitations section

This survey discusses the knowledge and risk behavior among students in Semey, Kazakhstan. It is a cross-sectional study, and although it might be of interest to do a follow-up study, it is beyond the scope of this project. It should be noted that the study was set in one city of Kazakhstan, owing to issues of access and practicality, and that the ethnic composition differs, with a larger Russian population in Semey than in cities in the south. It is therefore accepted that while our results are statistically significant they are not necessary representative of all of Kazakhstan. It is difficult to find other studies made in Kazakhstan, so many of the references cited are from other parts of Central Asia, the nearby Chinese province of Xinjiang, or other ex-Soviet countries. There are, therefore, some difficulties in comparing these studies appropriately. This study would benefit from a more thorough investigation of the students' attitudes and experiences of drug abuse and other stimulants. A larger number of students would also increase the statistical power of the findings.

## Conclusion

Among students in Semey, knowledge concerning routes of transmission and means of protection is good; however there are some difficulties in excluding routes of transmission and in understanding the difference between HIV and AIDS. There are various sources of information for young people, and most of our respondents had at least someone with whom they could talk about sexuality and HIV/AIDS. There is an acceptance of sexuality among young people and they can discuss issues of sexuality openly. One large risk factor for the spread of HIV/AIDS among young people in Kazakhstan is prostitution, amplified by stigmatization of the HIV positive and of homosexuals. Protective factors are the good knowledge about risks and protection, and opportunities to talk and gather information about sexuality and HIV/AIDS.

## Competing interests

The authors declare that they have no competing interests.

## Authors' contributions

RA conceived the study and participated in its design and helped to draft the manuscript. MH and LS carried out the fieldwork, handling questionnaires and interviews, performing the statistical analysis and the writing of the first draft. They worked in close cooperation with MU, who arranged all the practical matters on site. CA had input and ideas about the design and analysis of the study. All the authors read and approved the final manuscript.

## Pre-publication history

The pre-publication history for this paper can be accessed here:



## Supplementary Material

Additional file 1 **Approval from Ethics Committee, Semey, Kazakhstan** Click here for file
