# Supplementary material for: HIV/AIDS awareness and risk behavior among students in Semey, Kazakhstan: a cross-sectional survey
Source: BMC Int Health Hum Rights. 2008 Dec 16;8:14. doi: 10.1186/1472-698X-8-14 (PMC2630293; doi:10.1186/1472-698X-8-14)
Supplement: Supplementary file 1 [file 1472-698X-8-14-S1.rtf]

ҚАЗАҚСТАН РЕСПУБЛИКАСЫНЫҢ
ДЕНСАУЛЫҚ САҚТАУ МИНИСТРЛІГІ
РЕСПУБЛИКАЛЫҚ МЕМЛЕКЕТТІК
ҚАЗЫНАЛЫҚ КӘСІПОРЫН
СЕМЕЙ МЕМЛЕКЕТТІК
МЕДИЦИНА АКАДЕМИЯСЫ

МИНИСТЕРСТВО ЗДРАВООХРАНЕНИЯ
РЕСПУБЛИКИ КАЗАХСТАН
РЕСПУБЛИКАНСКОЕ ГОСУДАРСТВЕННОЕ
КАЗЕННОЕ ПРЕДПРИЯТИЕ
ГОСУДАРСТВЕННАЯ МЕДИЦИНСКАЯ
АКАДЕМИЯ ГОРОДА СЕМЕЙ

Абай Құнанбаев көшесі, 103, Семей қаласы
071400 Қазақстан Республикасы
т/факс: 52-22-51 т/факс: 56-97-55
e-mail: SGMA_mail@ok.kz

ул. Абая Кунанбаева, 103, г. Семей
071400 Республика Казахстан
т/факс: 52-22-51 т/факс: 56-97-55
e-mail: SGMA_mail@ok.kz

200 8 ж. «17» 06 №07-505 «17» 06 200 8 г.

BMC `International Health and Human Rights
The BioMed Central Editorial Team

According to position of Ethical Committee of Semipalatinsk State Medical Academy for research , " HIV/AIDS awareness and risk behavior among students in Kazakhstan: a cross sectional study" Marit Hansson, Leo Stockfelt, Marat Urazalin, Clas Ahlm and Rune Andersson Division of Infectious Diseases Sahlgrenska Academy at Geteborg University, Sweden, Division of Infectious Diseases, Department of Clinical Microbiology, Umea University Hospital, Sweden, Semipalatinsk State Medical Academy, Kazakhstan, lead by anonymous questionnaire the allowing certificate ethical is not required.

Head of the Ethical Committee
Professor: **R.Ivanova**

Rector Semipalatinsk State Medical Academy
Professor: **T.Rakhypbekov**

007719
